# The Emerging Role of Large Immunophilin FK506 Binding Protein 51 in Cancer

**DOI:** 10.2174/092986711798194333

**Published:** 2011-12

**Authors:** S Romano, A Sorrentino, AL Di Pace, G Nappo, C Mercogliano, MF Romano

**Affiliations:** Department of Biochemistry and Medical Biotechnology, Federico II University, Naples, Italy

**Keywords:** FKBP51, cancer, apoptosis, NF-κB, rapamycin.

## Abstract

FK506 binding protein 51 (FKBP51) is an immunophilin physiologically expressed in lymphocytes. Very recently, aberrant expression of this protein was found in melanoma; FKBP51 expression correlates with melanoma aggressiveness and is maximal in metastatic lesions. FKBP51 promotes NF-κB activation and is involved in the resistance to genotoxic agents, including anthracyclines and ionizing radiation. FKBP51 is a cochaperone with peptidyl-prolyl isomerase activity that regulates several biological processes through protein-protein interaction. There is increasing evidence that FKBP51 hyperexpression is associated with cancer and this protein has a relevant role in sustaining cell growth, malignancy, and resistance to therapy. There is also evidence that FKBP ligands are potent anticancer agents, in addition to their immunosuppressant activity. In particular, rapamycin and its analogs have shown antitumor activity across a variety of human cancers in clinical trials. Although, classically, rapamycin actions are ascribed to inhibition of mTOR, recent studies indicate FKBP51 is also an important molecular determinant of the drug’s anticancer activity. The aim of this article is to review the functions of FKBP51, especially in view of the recent findings that this protein is a potential oncogene when deregulated and a candidate target for signaling therapies against cancer.

## INTRODUCTION 

**Fig. (1):**
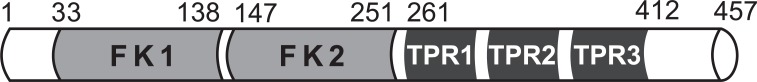
Structure of FKBP51. The N-terminal region of the FKBP51 contains two FKBP domains (FK1 and FK2). FK1 can catalyze the cis-trans conversion of peptidyl-prolyl-imide bonds in protein substrates. The C-terminal region of the FKBP51 includes three C-terminal TPR domains, responsible for protein protein interactions.

**Fig. (2):**
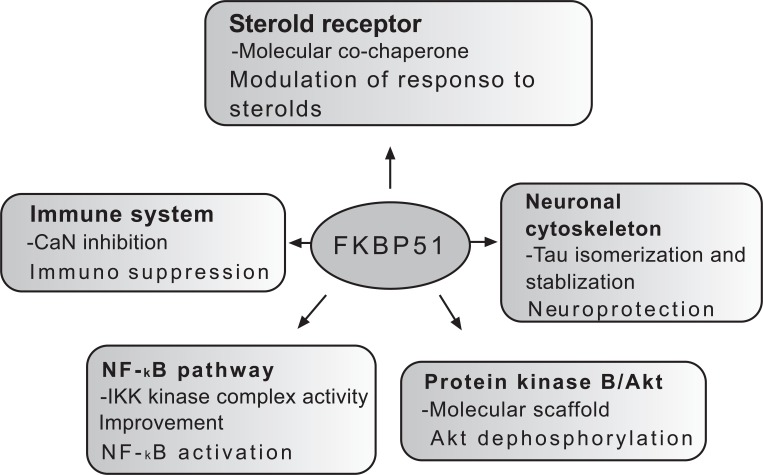
Functions of FKBP51. The multiple functions of FKBP51 can be dependent but also independent of the enzymatic activity.

FK506 binding proteins (FKBP) belong to the family of immunophilins, which also includes cyclophilins (Cyp) [1]. The designation “immunophilin” refers to the immunosuppressive character of Cyp/cyclosporin A and FKBP/FK506 complexes [2]. In addition to the capacity to bind to immunosuppressant drugs, immunophilins have another distinguishing property, namely peptidyl-prolyl cis-trans isomerase activity (PPIase) catalyzing the isomerization of peptidylprolyl imide bonds, from cis to trans, in protein substrates [1,2]. The enzymatic activity of immunophilins is inhibited by protein-ligand binding [1,2]. FKBP12 is the progenitor FKBP; the suffix designates the approximate molecular mass of this protein of 108 amino acids [1,2]. In humans, at least 15 FKBPs have been identified and named to reflect their molecular weights [3]. Family members of this ubiquitous enzyme class are found in abundance in virtually all organisms and subcellular compartments. Their amino acid sequences are highly conserved phylogenetically [2]. Organisms express many members of each family that encompass one or more PPIase domain, complemented with other functional polypeptide segments. These segments or domains include tetratricopeptide repeat (TPR) motifs involved in protein-protein interaction, EF-hand calcium-binding domain containing helix-loop-helix topology in which Ca2+ ions are coordinated within the loop, nucleic acid binding regions, transmembrane domain, and nuclear localization and endoplasmic reticulum signal sequences. These domains enable such proteins to perform a wide variety of cellular functions, including protein folding, improvement of kinase performance, receptor signaling, protein trafficking, and transcription [1-3]. There is evidence that FKBP ligands possess potent anticancer effects in addition to immunosuppressant activity [4-9]. In particular, rapamycin and its analogs, CCI-779 and RAD001, have shown antitumor activity across a variety of human cancers in clinical trials [4], and are approved for the treatment of renal carcinoma [10]. Most actions of rapamycin are mediated through specific inhibition of the mammalian target of rapamycin (mTOR) [4], which is the core of an evolutionarily conserved signaling pathway that controls the cell cycle in response to changing nutrient levels [11]. However, recent studies identify FKBP51 as an additional important molecular determinant of the drug’s anticancer activity [8,9,12]. FKBP51 structure (Fig. **1**) includes three C-terminal TPR domains, responsible for protein protein interactions with heat shock (chaperone) proteins HSP90 and HSP70 as well as with other proteins, including steroid receptors [1,3]. The N-terminal region of the FKBP51 contains two FKBP domains (FK1 and FK2) of which the more N- terminal one (FK1) can catalyze the cis-trans conversion of Xaa-Pro bonds, implying a role in protein folding [1,3]. The aim of this article is to review the functions of FKBP51 (schematically represented in Fig. **2**) with a focus on the emerging role of this protein as a tumor promoter.

## PHYSIOLOGICAL ROLES OF FKBP51

FKBP51 is capable of immunosuppression, mediated by calcineurin (CaN) inhibition, when complexed to FK506 [13]. CaN is a Ca2+/calmodulin-dependent serine-threonine phosphatase that regulates the clonal expansion of T cells after stimulation by an antigen, through activation of the nuclear factors of activated T lymphocytes (NFAT). NFAT proteins are phosphorylated and reside in the cytoplasm in resting cells; upon stimulation, they are dephosphorylated by CaN, translocate to the nucleus, and become transcriptionally active [14]. The NFAT group of transcription factors regulates production of interleukin 2 and a number of T cell specific activators [14], leading to immune response. Inhibition of CaN suppresses calcium-dependent early events of T-cell activation [13,14]. Among FKBPs, only FKBP12, FKBP12.6, and FKBP51 can mediate FK506 effects on CaN activity in human cells, whereas other FK506/FKBP complexes do not contribute to the inhibition of CaN protein phosphatase activity. Thereby, only FKBP12, FKBP12.6, and FKBP51 can be referred to as immunophilins [15].

FKBP51 interacts with glucocorticoid, androgen, estrogen, progestin and mineralocorticoid receptors [16-20]. The binding activity of steroid receptor to hormone is dependent on the ordered assembly of a mature receptor heterocomplex with various components of the molecular chaperone machinery, including Heat shock protein (Hsp) 90, the Hsp70-Hsp90-organizing protein Hop, FKBP52, FKBP51, Cyp40, and protein phosphatase PP5 [16]. Despite their similarities, the cochaperones FKBP51 and FKBP52 have distinctive properties for binding and assembling with Hsp90 in steroid receptor complexes [16,18]. In particular, FKBP52 and FKBP51 affect hormone binding to the GR in opposing manners. FKBP52 specifically enhances GR responsiveness to hormone [18]. The mechanism for potentiation is an increase in GR hormone-binding affinity that requires both the Hsp90-binding ability and the PPIase-mediated conformational changes in the ligand-binding domain [18]. FKBP51 blocks the potentiation mediated by FKBP52 [18]. Cortisol insensitivity is facilitated by a constitutive overexpression of FKBP51 [17]. Glucocorticoids up-regulate the gene for FKBP51, which provides a mechanism for desensitization of cells after an initial exposure to the hormone [17]. FKBP51 has the overall effect of reducing receptor transcriptional activity, with the exception of androgen receptor, whose transcriptional activity is increased by FKBP51 [21]. Genetic mutations leading to FKBP51 hyperexpression have been correlated to disfunction of stress hormone-regulating hypothalamic-pituitary-adrenal axis and major depressive disorders [22]. 

FKBP51 protects cells against oxidative stress [23]. Very recently, it has been demonstrated that FKBP51 is a major mitochondrial factor that undergoes nuclear-mitochondrial shuttling during the stress response and exerts antiapoptotic mechanisms. FKBP51 forms complexes in mitochondria with the glucocorticoid receptor and the Hsp90/Hsp70-based chaperone heterocomplex. Hsp90 inhibition promotes FKBP51 translocation from mitochondria to the nucleus in a reversible manner [23].

In the nervous system, FKBP51 regulates clearance of microtubule-associated protein tau and stabilizes microtubules [24]. Abnormal tau buildup leads to alterations of neuronal cytoskeleton, that is a common feature for a group of diseases termed tauopathies (the most common being Alzheimer's disease) [25, 26]. 

The FKBP51 homolog in Arabidopsis, PAS-1, plays a critical role in the growth and development of this organism [27]. In mammals, FKBP51 has a specialized role during cell division and is preferential expressed in mitotically active cells in the very early phases of differentiation [28-31]. FKBP51 is among the top candidates genes expressed during early mesenchymal differentiation into the three mesodermal lineages namely osteogenesis/chondrogenesis/adipogenesis [29]. At this stage, FKBP51 is co-expressed with ZNF145 [29], a gene whose abnormal rearrangement in chromosomal translocation t(11;17), causes acute promyelocytic leukemia [32]. ZNF145 is a member of the Krueppel C2H2-type zinc-finger protein family, it encodes a zinc finger transcription factor that interacts with a histone deacetylase and is involved in cell cycle progression [29,32]. 

## FKBP51 IN PRECANCEROUS DISEASE AND CANCER

The concept that FKBP51 is an essential factor for cell proliferation is also supported by studies on myeloproliferative disorders [33-35]. FKBP51 is overexpressed in idiopathic myelofibrosis [33], a chronic myeloproliferative disorder characterized by megakaryocyte hyperplasia and bone marrow fibrosis. Overexpression of FKBP51 in this disorder regulates the growth factor independence of megakaryocyte progenitors and induces an apoptotic resistance to cytokine deprivation mediated by the JAK/STAT5 pathway [34]. It has been shown that STAT5 is dephosphorylated on tyrosine residues more slowly in an FKBP51-overexpressing cell line. This condition sustains cell survival mechanisms, as suggested by the pro-apoptotic effect of a dominant negative variant of STAT5. The high pSTAT5 levels are, in turn, sustained by persistent JAK2 phosphorylation. The spontaneously megakaryocyte growth was abolished by the JAK2 inhibitor AG490. Furthermore, FKBP51 overexpression promotes fibrosis mediated by upregulation of TGF-β synthesis [35].

FKBP51 controls activation of NF-κB transcription factor that is involved in carcinogenesis, metastasis and resistance to anticancer therapy [36]. FKBP51 isomerase activity is important for the enzymatic function of the inhibitor κB kinase (IKK)α [37]. Bouwmeester et al. [37], by mapping the protein interaction network in TNF-α/NF-κB pathway found that FKBP51 co-purified with IKKα, IKKε, TGF-β Activated Kinase 1(TAK1) and Mitogen-activated protein kinase kinase kinase (MEKK1). Results from this study indicated that FKBP51 is a potential cofactor of multiple kinases. The interaction with IKKα was confirmed by co-immunoprecipitation assay and RNA interfering of FKBP51 strenghtened the importance of this immunophilin in the overall signaling process of NF-κB activation [37].

An additional mechanism that indirectly implicate FKBP51 in the regulation of NF-κB activation is through the inhibition of steroids response. Glucocorticoids are potent inhibitors of NF-κB activation, mediated by enhanced transcription of IκBα, that traps NF-κB in inactive cytoplasmic complexes [38]. In addition, the NF-κB subunit RelA, has been shown to physically interact with glucocorticoid receptor (GR) [39-41], which impairs the transactivating ability of this NF-κB subunit [42]. FKBP51 is part of the GR complex [16], its increased expression reduces hormone binding to receptor. Glucocorticoids up-regulate the gene for FKBP51, which serves to desensitisize cells after an initial exposure to the hormone [17]. Given the foregoing, a deregulated FKBP51 can virtually sustain NF-κB activation by reducing cortisol response.

In the canonical pathway of NF-κB activation, IKKα forms with IKKβ and a regulatory subunit IKKγ or NEMO the IKK kinase complex, that mediates the phosphorylation of IκB proteins in response to various stimuli [43]. Phosphorylated IκBs create a recognition signal for ubiquitinating enzymes, which mark IκBs for rapid 26S proteasomal degradation [43]. This process allows p50/RelA dimers to translocate to the nucleus, where they stimulate the expression of multiple target genes involved in immune response, inflammation, cell survival, anti-apoptosis, and cancerogenesis [44,45]. IKKα also works independent of the IKK complex. This occurs, for instance, in the alternative NF-κB pathway, which results in activation of p52/RelB heterodimers [46]. In this pathway, IKKα requires an upstream kinase, namely NF-κB-inducing kinase (NIK), and induces phosphorylation of p100 to produce mature p52 [46]. Furthermore, active IKKα phosphorylates cAMP response element-binding (CREB)-binding protein (CBP) at serine 1482 and serine 1386 [47]. Such phosphorylation enhances NF-κB-mediated gene expression and suppresses p53-mediated gene expression, thereby promoting cell proliferation and tumor growth [47].

The essential role of FKBP51 in the activation of NF-κB and NF-κB-regulated genes has been demonstrated, both in normal [48] and tumor cells [8,9,49], by gene silencing with short interfering RNA (siRNA). Defective activation of NF-κB in response to TNF-α was observed in vascular smooth muscle cells (VSMC) depleted of FKBP51 [48]. Similar results, defective NF-κB activation after FKBP51 depletion, were obtained in glioma [12], childhood acute lymphoblastic leukemia [9] and melanoma [8,49] after treatment with DNA damaging agents.

The Phosphatidylinositol 3-kinase (PI 3-kinase)/Protein kinase B (PkB or Akt) is an important pro-survival signaling pathway that is often deregulated in cancer. Recently, it has been found, in tumor cell lines, that FKBP51 acted as a scaffold to facilitate the interaction between Akt and PH domain leucine-rich repeat protein phosphatase (PHLPP), which mediates dephosphorylation of pAkt at S473 [50]. According to Pei *et al.* low levels of FKBP51 resulted in increased Akt phosphorylation and decreased chemosensitivity [50]. On the other hand, pAkt (S473) levels were not enhanced with downmodulation of FKBP51 in normal or cancerous tissue, in a melanoma xenograft mouse model [51]. In addition, a study conducted on samples of primary lymphatic leukemia and normal peripheral blood lymphocytes did not disclose any inverse correlation between FKBP51 and pAkt (S473) levels [51]. Thus, whether FKBP51 functions as Akt de-activator might depend on the tissue type and differences among pathways expressed in tumors. Mistafa *et al. *found that insulin increased binding of FKBP51 to pAkt (S473) in non-small cell lung cancer cells, A549, and that, in the presence of atorvastatin, the levels of pAkt bound to FKBP51 decreased in 5 min [52]. Although, the authors suggested a role of FKBP51 in atorvastatin-induced pAkt dephosphorylation, on the other hand, they demonstrated the involvement of CaN in the effect of atorvastatin. It was shown that atorvastatin stimulated nuclear localization of CaN, which in turn dephosphorylated Akt. FK506 prevented decrease of pAkt nuclear levels by atorvastatin [52]. The findings of Mistafa *et al. *are not in concert with the notion that FKBP51 is an immunophilin capable of CaN inhibition [13,53]. According to this capability, overexpression of FKBP51 is expected to induce a marked inhibition of calcineurin activity, that should impair the de-phosphorylation of Akt. In conclusion, the role of FKBP51 in Akt dephosphorylation remains not defined in the paper of Mistafa. 

Iperexpression of FKBP51 has been documented in several human cancers. Enhanced FKBP51 expression is associated with apoptosis resistance and enhanced proliferation in gliomas [12]. A study of specimens from 192 patients, including glioblastoma multiforme, oligodendrogliomas, astrocytomas, and mixed gliomas, showed the FKBP51 expression level correlated with grading [12]. The study of patient survival showed that the expression of FKBP51 correlated with overall glioblastoma patient survival rates; that is, the glioblastoma patients with high levels of FKBP51 had shorter survival than those with intermediate levels [12]. Moreover, using both hyperexpression and knock-down approaches, it was shown that FKBP51 proliferative and anti-apoptotic properties in gliomas were mediated by NF-κB activation [12]. A protective role of FKBP51 on apoptosis was also described in retinal tumor cells [54]. 

Increased levels of FKBP51 are also associated with tumor aggressiveness in melanoma [8,49]. A study of specimens from 80 melanoma patients showed that FKBP51 was expressed in all cutaneous malignant melanomas analyzed but not in normal skin; expression was higher in melanocytes during the vertical growth phase compared to the radial growth phase and correlated with the thickness of the tumor lesion. FKBP51 expression was maximal in metastatic cutaneous lesions [49]. Interestingly, a remarkable FKBP51 level, even higher when compared to that of differentiated tumor cells, was found in melanoma cancer stem/initiating cells [49]. Experiments with FKBP51 knock-down showed the protein played a relevant role in resistance to chemo- [9] and radio-therapy-induced apoptosis [49]. 

In prostate cancer, FKBP51 is part of a superchaperone complex that includes androgen receptor (AR) and androgen [55]. Depleting FKBP51 levels by short hairpin RNA reduced the transcript levels of genes regulated by AR and androgen, suggesting the importance of this immunophilin in determining the ligand-binding competence and transcription function of AR [55]. In prostate cancer, FKBP51 is upregulated in association with cyclophilin Cyp40 [6]. In androgen-dependent tumor cell lines, FKBP51 hyperexpression increased androgen receptor transcriptional activity in the presence and absence of androgens; whereas, knockdown of FKBP51 dramatically decreased androgen dependent gene transcription and proliferation. In androgen-independent prostate cancer, FKBP51 was also found hyperexpressed [6]. Most intriguingly, a very recent paper implicates IKKα, whose function is enhanced by FKBP51 co-chaperone [37], in the androgen independent growth of prostate cancer [56]. 

An immunohistochemistry study of expression of FKBP51 in 50 tumoral samples including breast, lung, pancreas, ovary, and prostate (10 samples for each tumor), and a comparable number of normal tissue samples showed an intense signal in 38 out of 50 tumors analyzed, whereas normal tissues of the same histotypes showed a weak/absent immunohistochemical signal [51]. Data on prostate cancer were in agreement with those of Periyasamy *et al. *[6]. All prostate cancer samples analyzed displayed an intense FKBP51 immunochemical signal, which was restricted to the tumor. A similar intense immunoreactivity was found in ovary cancer samples. Intriguingly, in lung cancer, FKBP51 immunoreactivity was nuclear. The 12 tumor samples with low/negative immunohistochemistry were the 10 breast cancer samples and 2 out of 10 pancreatic tumors. Interestingly, these two pancreatic tumors belonged to the well-differentiated histotype (G1). Measurement of FKBP51 mRNA levels in deparaffinized tissues using real-time PCR confirmed the immunohistochemistry results [51].

## FKBP51 AS A TARGET FOR ANTICANCER THERAPY

The first evidence supporting the involvement of FKBP51 in the resistance to cancer therapies came from mechanistic studies of the antitumor activity of rapamycin [8,9]. Rapamycin is a conventional immunosuppressant agent with anticancer properties [4]. Both immunosuppression and anticancer effects of rapamycin are classically ascribed to inhibition of mTOR. The rapamycin/FKBP complex interacts with the FKBP-rapamycin binding domain in mTOR, adjacent to the catalytic kinase domain, and blocks its function [57]. mTOR is a member of the ataxia-telangiectasia-mutated (ATM) family of kinases that functions as a checkpoint for nutritional status in G1 [58]. It is a serine-threonine kinase that, upon activation, phosphorylates the ribosomal protein S6 kinase [59] and the eukaryotic initiation factor 4E binding protein 1 (4E-BPI) [60], which promotes ribosome biogenesis and induces the translation of proteins involved in cell cycle progression and proliferation [59-61]. mTOR is activated downstream in the phosphatidylinositol 3 kinase (PI3k), protein kinase B (PkB or Akt) signaling pathway [62,63]. Many genomic aberrations in human cancers result in activation of this pathway, which alters the control of survival and leads to defective apoptosis [64]. Generally, such genomic aberrations involve deletions of the tumor suppressor gene PTEN, the inositol 3-phosphatase and tensin homologue, which dephosphorylates phosphoinositides at the D3 position, thereby terminating the PI3k/Akt cascade [65]. A number of rapamycin analogs have been developed over recent years. They include CCI-779, RAD-001, and AP23573 [66] and their major application is in the treatment of PTEN-deficient tumors [67]. 

In a study conducted in melanoma, a tumor in which PTEN is often mutated [68], rapamycin markedly enhanced doxorubicin-induced apoptosis [8]. However, contrary to expectations, the apoptosis sensitizing effect of rapamycin appeared to be independent of blockage of the PI3k/Akt/mTOR pathway and was instead associated with inhibition of NF-κB signaling. Rapamycin, but not wortmannin, a PI3k inhibitor, induced a block of the IKK kinase phosphorylating ability [8]. The effect of rapamycin on NF-κB was reproduced using FKBP51 siRNA, which depleted the cell of this protein. In accordance, the NF-κB regulated genes, Bcl-2 [69] and c-IAP1 [70], were decreased; these genes have been implicated in tumor cell resistance to doxorubicin [71,72]. The finding that rapamycin can sensitize cancer cells to apoptosis, independent of mTOR inhibition, was confirmed in childhood acute lymphoblastic leukemia (ALL) [9]. This cancer comprises a heterogeneous variety of subtypes. In most of these leukemias, blasts are sensitive to the apoptosis-inducing effect of rapamycin because of constitutive activation of the PI3k/Akt pathway, which sustains blast survival [73]. Rapamycin increased doxorubicin-induced cell death, even in samples that did not respond to PI3k inhibition, while wortmannin was unable to cooperate with the anthracycline drug. The apoptosis sensitizing effect of rapamycin was counteracted by hyperexpression of the NF-κB RelA subunit in leukemic cells [9]. Increased cytotoxicity to doxorubicin was observed in leukemic cells depleted of FKBP51 [9]. These results support the conclusion that rapamycin may also be effective against neoplasias that express the tumor suppressor PTEN. Consistent with this conclusion, rapamycin retained the capacity to cooperate with NF-κB-inducing chemotherapeutics in PTEN reconstituted tumors [74].

The role of FKBP51 in the resistance of tumors to cell death has been extensively studied in melanoma [8,49]. In this tumor, ionizing radiation activates autophagy but not apoptosis and increases levels of the X-linked inhibitor of caspases, xIAP [49]. The formation of autophagic vacuoles was visualized as soon as after 6 hours from ionizing radiation exposure. This was preceded by an increase of Beclin-1, a protein that plays an essential role in the formation of initial autophagosomes, as a component in the class III PI3k complex [75]. In addition, confocal microscopy showed that Bax, the lethal effector of the mitochondrion-dependent cell death program, was included in autophagic vacuoles. This supports the hypothesis that an autophagic degradation of Bax prevents from induction of apoptosis. The up-regulation of xIAP by ionizing radiation also played a role in the inhibition of apoptosis in irradiated melanoma. In fact, when xIAP expression was silenced, ionizing radiation stimulated caspase activation and cell death. This study emphasizes the central role of FKBP51 in the activation of ionizing radiation-induced NF-κB, which in turn inhibits apoptosis by stimulating xIAP and promoting autophagy [49]. FKBP51 knock-down efficiently overcomes the blockage of apoptotic machinery. FKBP51-silenced melanoma exhibited reduced clonogenic potential after irradiation, compared to non-silenced melanoma; moreover, melanoma xenografts implanted in nude mice showed unequivocal and extensive apoptosis provoked by a single dose of siRNA prior to irradiation [49]. Interestingly, ABCG2+ melanoma cells, that possess the capacity to self-renew and generate the heterogeneous cancer cells [76,77], contain intracytoplasmic FKBP51 at higher levels than ABCG2- cells [49]. These melanoma cancer stem cells are killed by ionizing radiation after pre-treatment with siRNA, providing the basis for radiocurability of the tumor [78]. 

## CONCLUSION

FKBP51 is a protein with a progressively emerging role in cancer biology. Although its function is still far from being fully elucidated, there are clear data suggesting this immunophilin plays an active role in cell proliferation in both the physiologic conditions of cell growth and differentiation [28-31] and in pre-neoplastic [33-35] and neoplastic diseases [6,12]. Furthermore, it is involved in resistance to cell death [8,9,12,49]. Several lines of evidence support the conclusion that FKBP51 is a promising molecular therapeutic target in cancer [6,8,9,12,49]. Notably, FKBP51 blockage mediates some of the pro-apoptotic effects of rapamycin [8,9,12,16,49]. Rapamycin targets two crucial signaling pathways involved in cell survival and chemoresistance of cancer cell, namely PI3k/Akt through mTOR and NF-κB through FKBP51. So far, rapamycin derivatives have been created to increase the bioavailability of the drug and strengthen the interaction with mTOR. It is to note that mTOR is a negative regulator of autophagy [79]. Separating FKBP51 inhibition from mTOR inhibition in anticancer therapy may sometimes be useful, according to recent studies that propose a role for autophagy in promoting cancer survival and enhancing the threshold for apoptosis [80]. In this view, small molecules specifically targeting FKBP51 may result, in some cases, more efficacious than rapamycin as anticancer agents. 

Finally, increased FKBP51 seems to be a common denominator between depression and cancer [81]. In the future, a full elucidation of the effect of deregulated FKBP51 on cell omeostasis and eventual role in epigenetic changes in the genome, that concur with cancer development, may provide the molecular basis of this long-observed linkage between depression and the onset of cancer.

## ABBREVIATIONS

ABCG2: = ATP-binding cassette sub-family G member 2
Akt: = protein kinase B
CaN: = calcineurin
CBP: = CREB-binding protein
CREB: = cAMP response element-binding
Cyp: = Cyclophilin
FKBP: = FK506 binding protein
GR: = glucocorticoid receptor
Hsp: = Heat shock protein
IKK: = inhibitor κB kinase
mTOR: = mammalian target of rapamycin
NF-κB: = nuclear factor of κ light-chain-enhancer of activated B cells
NFAT: = nuclear factors of activated T lymphocytes
NIK: = NF-κB-inducing kinase
PHLPP PH: = domain leucine-rich repeat protein phosphatase
PI3k: = phosphatidylinositol 3 kinase
PPIase: = peptidyl-prolyl cis-trans isomerase activity
siRNA: = short interfering RNA
TGF-β: = Transforming Growth Factor-β
VSMC: = vascular smooth muscle cell
xIAP: = X-linked inhibitor of caspases
ZNF145: = zinc-finger protein 145
